# Outcome of Hydroxyurea Use in SCD and Evaluation of Patients’ Perception and Experience in Nigeria

**DOI:** 10.3389/fgene.2022.826132

**Published:** 2022-03-24

**Authors:** Reuben Ikechukwu Chianumba, Akinyemi O. D. Ofakunrin, Jack Morrice, Olaniyi Olanrewaju, Oluseyi Oniyangi, Aisha Kuliya-Gwarzo, Uche Nnebe-Agumadu, Hezekiah Alkali Isa, Obiageli Eunice Nnodu

**Affiliations:** ^1^ Centre of Excellence for Sickle Cell Disease Research and Training, University of Abuja, Abuja, Nigeria; ^2^ Department of Paediatrics, Jos University Teaching Hospital, Jos, Nigeria; ^3^ Division of Human Genetics, Department of Pathology, Faculty of Health Sciences, University of Cape Town, Cape Town, South Africa; ^4^ Irua Specialist Teaching Hospital, Irrua, Nigeria; ^5^ Department of Paediatrics, National Hospital, Abuja, Nigeria; ^6^ Bayero University Kano/Aminu Kano University Teaching Hospital, Kano, Nigeria; ^7^ Department of Paediatrics, University of Abuja Teaching Hospital, Gwagwalada, Nigeria; ^8^ Department of Haematology and Blood Transfusion, College of Health Sciences, University of Abuja, Abuja, Nigeria

**Keywords:** sickle cell disease, patients perception, patients experience, Nigeria, hydroxyurea

## Abstract

**Introduction:** Hydroxyurea (HU) has been shown to be beneficial in the management of sickle cell disease (SCD) as it improves treatment outcomes. However, despite the benefits of HU, its uptake among SCD patients in Nigeria remains low.

**Objective:** This study aimed to assess the perception and experience of patients with SCD in Nigeria who are using or had used HU, thereby informing and promoting its use.

**Methodology:** A multi-centre, cross-sectional study was conducted among 378 SCD patients aged 1–53 years who have enrolled on Sickle Pan African Research Consortium (SPARCO) registry as HU users. The SPARCO project was funded by the National Institutes of Health (NIH) to establish a sickle cell disease (SCD) registry, strengthen skills and plan research in three African countries. The Nigerian SPARCO registry had 6453 SCD patients at the time of this report with <15% of this population on HU. Data on sociodemographics, perception and experience about HU use were obtained and analysed using descriptive statistics.

**Findings:** Out of the 378 participants, 339 (89.7%) were using HU while 39 (10.3%) had stopped using HU at the time of the study. 281 (74.3%) found HU expensive, while 194 (51.3%) reported none to minimal side effects while using HU. Among patients that stopped HU, cost (59%) and availability (51.3%) were the commonest reasons for discontinuing the drug. Furthermore, 347 (92.5%) had fewer pain crises, 173 (84.8%) had a fewer need for blood transfusion, 145 (86.3%) had improved PCV and 318 (84.6%) had fewer hospital admissions. Finally, the study also showed that 322 (85.2%) respondents would recommend the drug to other patients, whereas 14 respondents (3.7%) would not. Mean corpuscular volume (MCV) and fetal hemoglobin (HbF) levels were not collected in this study and may have improved findings.

**Conclusion:** This study showed that the majority of the SCD patients had good perception and experience with the use of HU while a few had to stop the medication mostly on account of cost and availability. Patients’ based advocacy could be leveraged to improve HU uptake while more efforts are needed to ensure that it is readily available and affordable.

## Introduction

Sickle Cell Disease (SCD) is prevalent in many low and middle-income countries (LMICs) especially in sub-Saharan Africa (SSA). Several studies have reported that more than 75% of the estimated 300,000 individuals born with SCD annually are within the SSA (Adewoyin et al., 2017; Adegoke et al., 2014; Galadanci et al., 2014). This region is characterized by poor health-seeking behaviour, inadequate health amenities, very low or nonexistent neonatal screening programs for SCD, and insufficient government policies to improve health care for SCD patients (Adegoke et al., 2014). These have contributed to the poor survival outcomes and quality of life (QOL) for SCD patients within the region. SCD accounts for the greater proportion of morbidity and mortality of infants and children under 5 years (U5). In Nigeria, more than 100,000 SCD births are estimated annually. Of these, approximately 70% are estimated to die before their fifth birthday (Piel et al., 2013; Nnodu et al., 2021). Other than the high mortality rate, the quality of life of individuals living with this disease is far below normal (Piel et al., 2013; Makani et al., 2013).

This condition is a life-long illness marked by events of painful crisis which require health maintenance and sometimes hospitalization. Some medications and lifestyles of the patients have proven to be very effective in the management of SCD by reducing the symptoms and improving the disease outcome and QOL (Brandow and Panepinto, 2010; Nebor et al., 2013; Badawy et al., 2017). For instance, prophylaxis with immunizations, regular use of anti-malarial medications, antibiotics, and HU have shown to be effective even in resource-limited settings (Nebor et al., 2013)^;^ Makani et al., 2013; Ware, 2015).

HU has been shown by several studies to be beneficial in the management of SCD; it improves treatment outcomes in addition to reducing the financial burden of the disease on families (Badawy et al., 2017; Crego et al., 2020; Smith et al., 2011; Wang et al., 2013). However, despite the benefits of HU in the management of SCD, its utilization among health care providers, caregivers and patients has remained low. Some documented reasons for this low level of utilization include side effects, cost, availability, poor awareness about the use of HU, adherence, or unavailability of guidelines on the administration and monitoring of HU (Thornburg et al., 2010)^;^ Yawn et al., 2014; OD Ofakunrin et al., 2021; Adegoke et al., 2014; Adewoyin et al., 2017; Badawy et al., 2017; Adeyemo et al., 2019; Brandow and Panepinto, 2010). In Nigeria, the Sickle Pan African Research Consortium (SPARCO), which was established to maintain a registry of 13,000 SCD patients for 4 years across the three participating countries, Ghana, Nigeria and Tanzania, was able to enrol 6,453 patients into the Nigeria registry using the Research Electronic Data Capture software (REDCap). Of this number, less than 15% had used HU (Isa et al., 2020; Makani, 2021)

In view of this, we hypothesized that evaluating the perception and experience of patients with SCD who are using or had used HU could help in gaining insight into the possible barriers and promoters/facilitators of HU uptake, hence this study.

## Methodology

### Study Design

**FIGURE 1:**
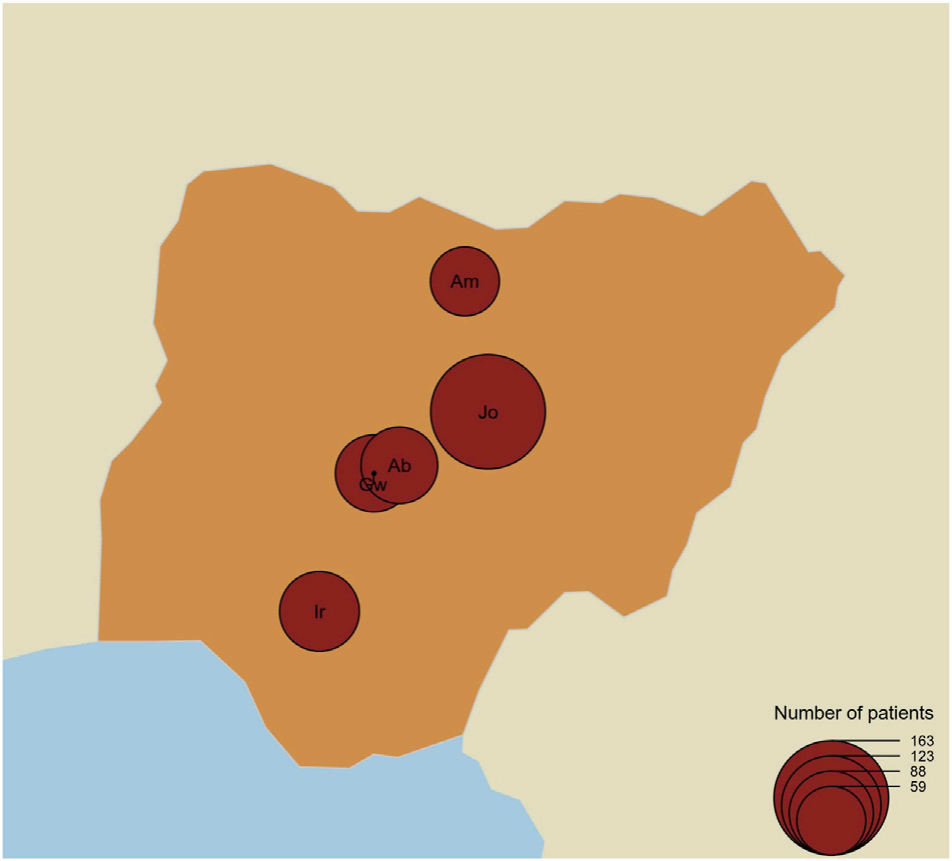
Map of Nigeria showing the number of patients per centre and locations of the study centre. The map shows the location of the study centres and the size (black circles) of patients recruited for the study per centre. Gw = University of Abuja Teaching Hospital, Gwagwalada, Jo = Jos University Teaching Hospital, Jos, Am=Aminu Kano Teaching Hospital, Kano, Ab = National Hospital Abuja, Abuja, and Ir=Irrua Specialist Teaching Hospital, Edo.

This study employs both a cross-sectional and retrospective study approach for the participation of SCD patients who reported the use of HU in the treatment of SCD in the SPARCO Nigeria SCD registry. At the time of this study, a total of 6,453 patients, from 20 health facilities, were enrolled in the SPARCO REDCap Registry; 917 (14.2%) of these patients reported the use of HU. Considering the study population estimates, we selected five tertiary health facilities which accounted for most of the patients using HU in the database. University of Abuja Teaching Hospital, Gwagwalada, Federal Capital Territory (FCT), Jos University Teaching Hospital, Jos Plateau State, Aminu Kano Teaching Hospital, Kano State, National Hospital Abuja, Abuja FCT, and Irrua Specialist Teaching Hospital, Edo State accounted for 70.6% (647) of the number of patients who reported the use of HU in the registry (Figure 1).

### Inclusion Criteria

Participants of this study had to be SCD patients registered in the SPARCO REDCap database, with a history of the use of HU, whose telephone number was valid and could be reached during the period of data collection, and who attended clinics such that their medical record folders could be accessed.

### Exclusion Criteria

SCD patients who had no history of the use of HU, who could not be reached.

### Sampling and Sample Size

Using the known population size of 917 patients on HU in the registry, confidence interval of 95%, the margin of error of 5%, population proportion of 50%, we were able to reach a minimum sample size of 271 for this study. We also adopted the 50% proportion size across the participating sites to generate the minimum sample size per site (Table 1). We then used a simple random sampling technique to recruit the patients. We went further to design a structured interviewer-administered multiple-choice questions using the REDCap data collection tool for the study (https://redcap.uniabuja.edu.ng/surveys/?s=YH9HH9FLTT). We contacted these patients via telephone calls or had a face to face interaction with those who were scheduled for clinic visits during the time of data collection. Since they had previously consented to participate in the SPARCO project, verbal consent was obtained from respondents. Furthermore, we extracted demographic data and other relevant information for the participants from the registry, this was analyzed alongside the data from the phone interview to inform the outcome of this study (Table 1).

**TABLE 1:** Patient distribution across participating centres

Hospital Name	Patients in Database =N	Patients on HU n(% of N)	Minimum sample size based on 50% proportion	Patients recruited U=378 n(% of U)
Jos University Teaching Hospital	505	310 (61.4)	155	163 (43.1)
National Hospital Abuja	247	80 (32.4)	40	71 (18.8)
University of Abuja Teaching Hospital, Gwagwalada	911	97 (10.6)	48.5	62 (16.4)
Irrua Specialist Hospital	99	90 (90.9)	45	46 (12.2)
Aminu Kano Teaching Hospital	222	70 (31.5)	35	36 (9.5)

Values represented in numbers and percentages, n=number, U=number of patients included in the study.

### Data Analysis

We analysed responses from participants asked if they were still using or had stopped HU treatment, how long they used HU, what dose they started with or were using at the time of the report, and how often they took the medication. We also analysed responses concerning pain experience using HU, questions like frequency of pain crises before and during HU treatment, SCD complications and frequency of hospital admission were asked to the participants. Lastly, we looked at responses pertaining to blood transfusion history, PCV values before and during the treatment with HU, and perception on cost, availability and side effects using HU. The data collected were processed and analysed using the R statistical software, and the tidyverse R package. Missing values were imputed using a random forest imputation algorithm implemented in the random forest R package. All binary outcomes were modelled with logistic regression using the multinomial distribution. We considered *p*-values statistically significant if they were below 0.05. All plots were developed with ggplot2 and the map of Nigeria was created using the R package Cartography.

## Results

A total of 378 patients were interviewed between April 18, 2021, and 9 July 2021, for this study. Seventy-four (16.5%) of these patients received care from the University of Abuja Teaching Hospital, Gwagwalada FCT, 163 (36.4%) from Jos University Teaching Hospital, Jos Plateau State, 59 (13.2%) from Aminu Kano Teaching Hospital, Kano State, 73 (16.3%) from National Hospital Abuja, FCT and 79 (17.6%) Irrua Specialist Teaching Hospital, Edo state (Table 1). The patients comprised 212 (56.1%) males and 166 (43.9%) females between the ages of 1–53 years and a median age of 11 years (Table 2). At the time of the study, 339 (89.7%) patients were on HU while 39 (10.3%) had stopped using the drug. We found also that 322 (95.0%) patients adhered to the daily use of HU, while others who did not adhere to the prescription, either took the drug weekly, three times a week or whenever the drug was available.

**TABLE 2:** Characteristics for participants

Variable	Frequency	Percentage
Age (years)	—	—
0–15	273	72.2
16–30	97	25.7
31–45	7	1.9
>45	1	0.3
Median age (years) = 11	—	—
**Sex**	**Frequency**	**Percentage**
Male	212	56.1
Female	166	43.9
History of HU use	—	—
Currently on HU	339	89.7
Stopped HU	39	10.3
**Duration of HU use (years)**	**Frequency**	**Percentage**
≤1	60	15.9
>1–5	238	63.0
>5	33	8.7
Not Sure	47	12.4
**Start dosage of HU (mg)**	**Frequency**	**Percentage**
250	98	28.9
500	115	33.9
1000	10	2.9
1500	1	0.3
Others	106	31.3
Mean (SD)=640.5 (269.5), Min= 100 mg, Median= 600 mg, Max= 1500 mg	—	—
**Current dosage of HU (mg)**	**Frequency**	**Percentage**
250	39	11.8
500	87	26.4
1000	75	22.7
1500	2	0.6
Others	127	38.5
Mean (SD)= 638.4 (270.0), Min= 150 mg, Median= 600 mg, Max= 1500 mg	—	—
Minimum dose of HU = 7 mg/kg	—	—
Median dose of HU = 21 mg/kg	—	—
Maximum dose of HU = 35 mg/kg	—	—
**Frequency of HU use**	**Frequency**	**Percentage**
Daily	322	85.2
Weekly	2	0.5
Others	6	1.6
Missing	48	12.7

### Perception of the Use of Hydroxyurea

**FIGURE 2:**
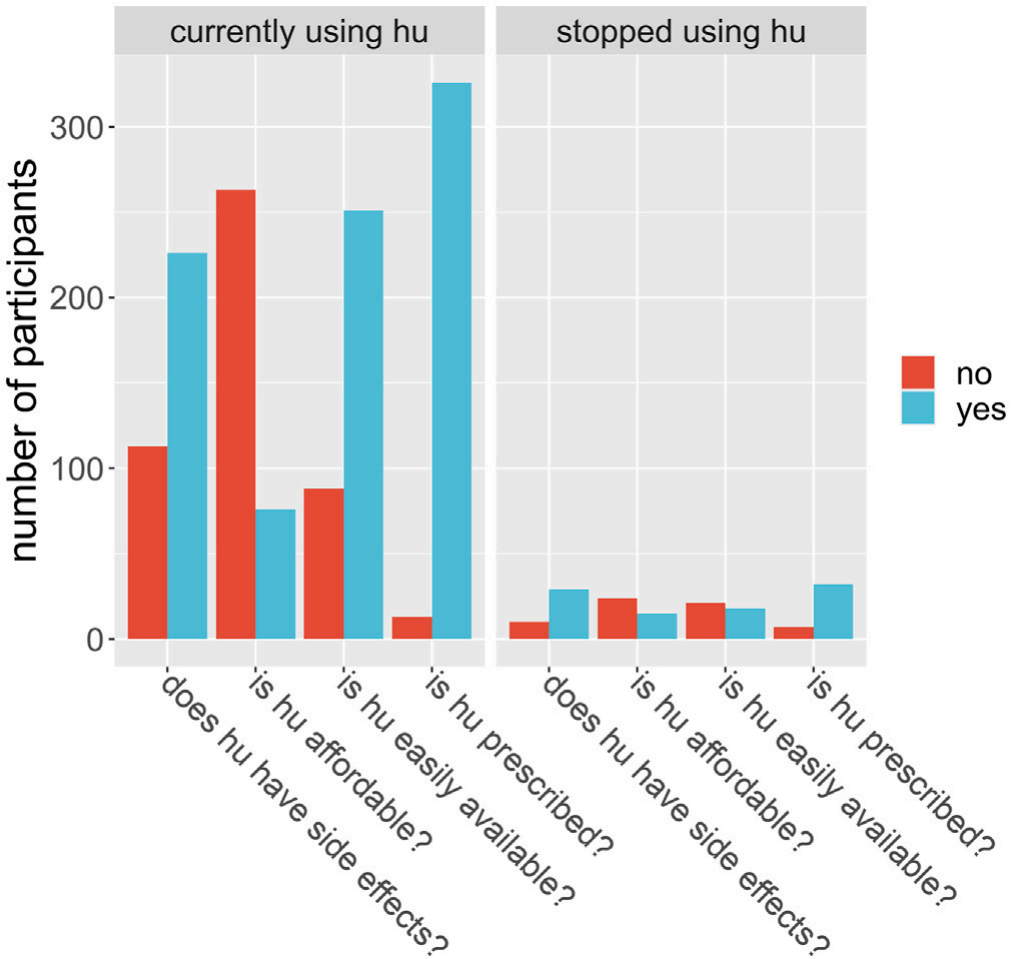
Participants were asked a set of 4 questions, shown on the x-axis, around their perceptions of hydroxyurea (hu). The number of participants (y-axis) answering yes (blue) or no (red) are shown by the bars, for those who are currently using hu (left plot) and for those who did use hu but had stopped at the time of the questionnaire (right plot).

**FIGURE 3:**
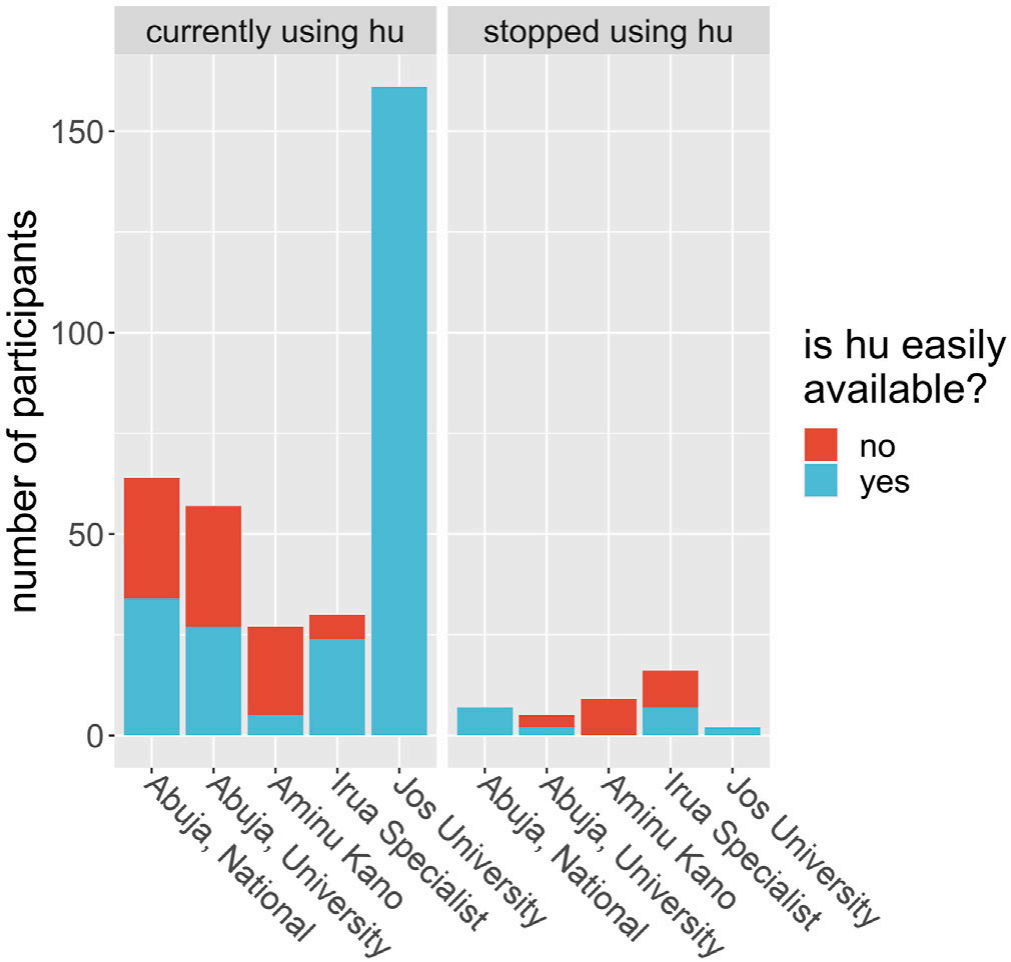
Study participants were asked if hydroxyurea (hu) was easily available to them at the time of the study. Each bar shows the number of participants at each study center (x-axis) who responded yes, it is easily available (blue) or no, it is not easily available (red) given that they were either currently using hu (left plot) or did use hu but had stopped at the time of the questionnaire (right plot). The blue and red bars are stacked on top of each other, so the height of each bar represents the total number of patients at that hospital.

We evaluated patients’ perception of the use of HU based on if they found the drug affordable, accessible, without side effects and if care providers prescribed the drug routinely. We observed that doctor’s advice significantly influenced whether or not the patient will use the prescription. Of all the 378 participants interviewed, 281 (74.3%) said they believed HU was expensive. For the subgroup of participants who were using HU at the time of the interview, 258 out of 339 said they believed the drug was expensive; for the rest of the participants, those who were using HU at some time in the past but have since stopped, 23 out of 39 (59%) said they believed it was expensive. Is this difference in opinion between users and nonusers significant? A chi-square test is illuminating here. Take the null hypothesis to be that a person who believes HU is affordable is no more or less likely to continue to use HU over a person who does not believe it is affordable. Using the data, and a Pearson’s chi-square test with Yates’ continuity correction, we find the probability of obtaining these proportions given the null hypothesis is 0.034. Therefore, we found strong evidence that cost is a factor in determining who will and will not use HU. Similarly, we found that accessibility of HU significantly influenced if the patient continues to use the drug (Table 3; Figure 2). Is a participant’s local hospital an important factor in their decision to continue to use HU? From Figure 3 we see that this may be true. For example, at Jos University teaching hospital 161 out of 163 patients (99%) were using the drug whereas at Irrua Specialist 30 out of 46 (65%) were using it. Assuming a null hypothesis that a patient at Jos is equally likely to continue to use HU as one who is at another hospital in the study, a simple chi-square test gives us a *p*-value of less than 0.001 of obtaining the same data given this null hypothesis is true. We therefore find strong evidence that the patient’s local hospital can be an influencing factor in their decision to continue HU treatment. Why might the hospital itself be an influencing factor in this decision? We propose that HU is not equally available in the different hospitals across Nigeria and that this difference may account for some of this influence. For example, hospitals may not always have enough stores of the drug to meet their demand, or they may not receive shipments as regularly as in other areas. To test this proposal, we looked at how a participant’s perception of the availability of the drug depends on their local hospital. At Jos, we find that 163 of 163 participants (100%) said they believed that HU was easily available, whereas, for participants at all other hospitals combined, only 105 out of 215 participants (49%) said they believed it was easily available. A chi-square test is not advisable in this instance, but it is clear that there is a correlation between the availability of the drug and its continued use, in this example. Furthermore, we were able to deduce that the reason most significantly associated with continued HU use as given by the participants was that the doctor prescribed it and that it is easily available. Finally, side effects did not significantly influence this outcome as most participants reported little or no side effects (Table 3).

**TABLE 3:** Perception on the use of hydroxyurea

Perception	Currently using HU	Stopped using HU	*P-*value
HU is affordable	—	—	—
True	57 (16.8)	12 (30.8)	0.034
False	258 (76.1)	23 (59.0)	—
Don’t know	14 (4.1)	3 (7.7)	—
Missing	10 (2.9)	1 (2.6)	—
HU is available	—	—	—
True	247 (72.9)	16 (41.0)	0.0329
False	66 (19.5)	20 (51.3)	—
Don’t know	16 (4.7)	2 (5.1)	—
Missing	10 (2.9)	1 (2.6)	—
HU has no side effect	—	—	—
True	167 (49.3)	27 (69.2)	<0.01
False	83 (24.5)	9 (23.1)	—
Don’t know	79 (23.3)	2 (5.1)	—
Missing	10 (2.9)	1 (2.6)	—
Is HU prescribed	—	—	—
True	320 (94.4)	30 (76.9)	<0.01
False	2 (0.6)	6 (15.4)	—
Don’t know	7 (2.1)	2 (5.1)	—
Missing	10 (2.9)	1 (2.6)	—
Would you recommend HU	—	—	—
True	306 (90.3)	16 (41.0)	—
False	10 (2.9)	4 (10.3)	—
Don’t know	20 (5.9)	19 (48.7)	—
Missing	3 (0.9)	—	—

### Experience With the Use of Hydroxyurea

**FIGURE 4:**
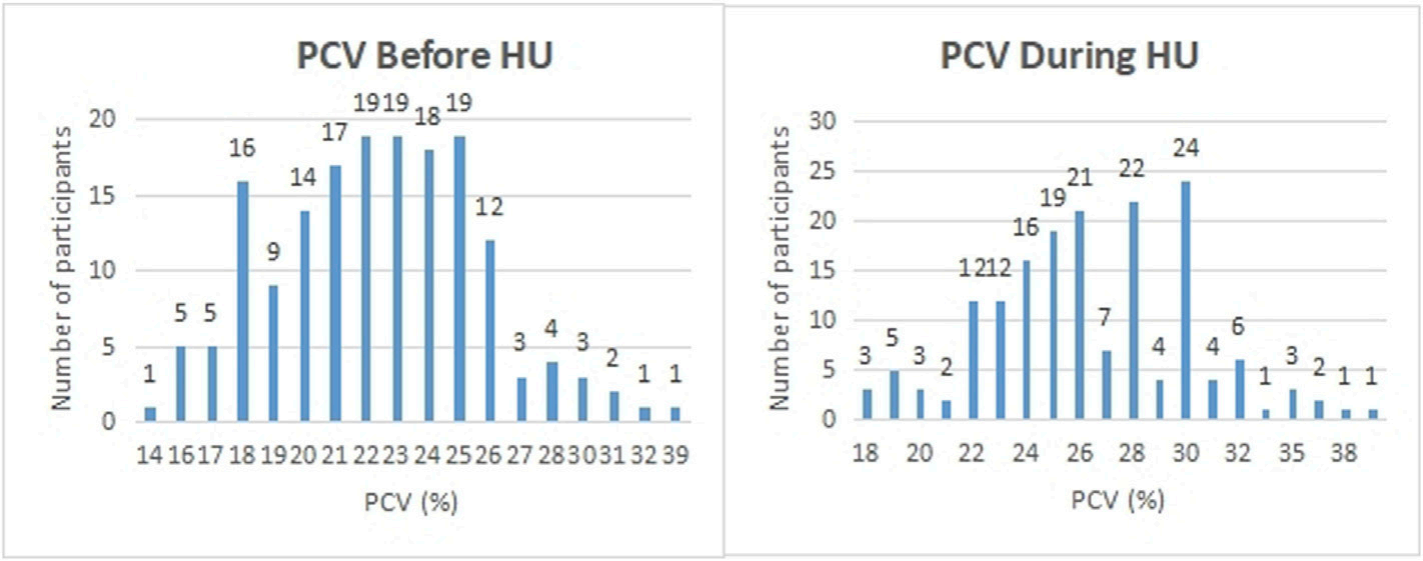
Participants were asked what their PCV was before and during the period of hydroxyurea treatment. The PCV (%) reported by participants are shown on the x-axis while the number of participants on the y-axis.

**FIGURE 5:**
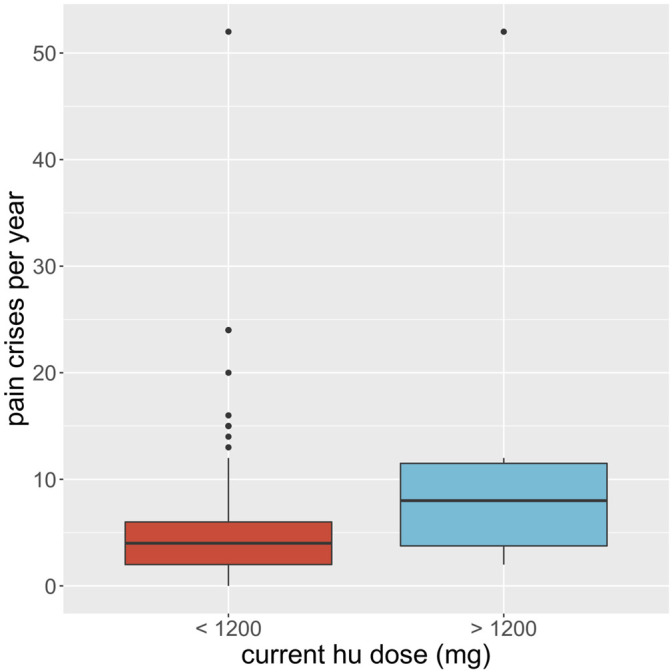
Boxplot of the average recalled number of painful crises a participant experiences per year (y-axis) is compared for participants on regular hydroxyurea (hu) doses, less than 1200 mg per week (red), to those on exceptionally high doses, more than or equal to 1200 mg per week (blue). Black dots show the outlying participant responses.

**FIGURE 6:**
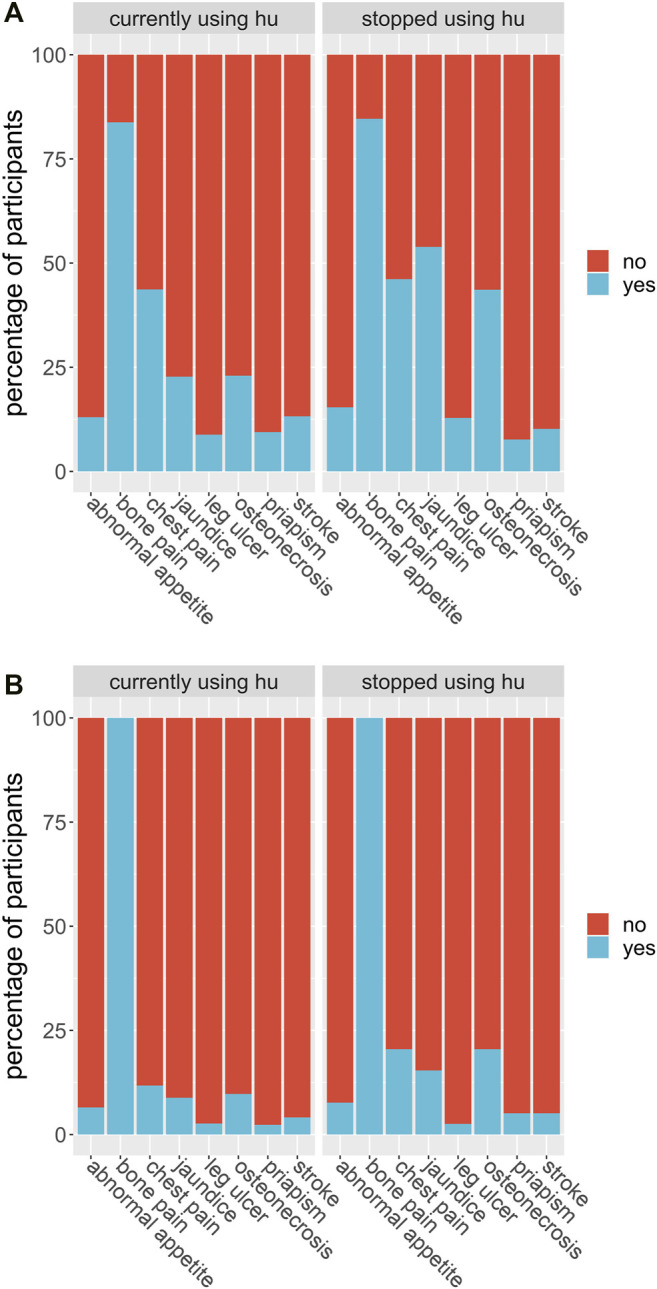
**(A)** Participants were asked if they suffered from each of a list of known sickle cell disease complications *before* they began their hydroxyurea treatment. The proportion of participants out of the entire study group who answered yes, they did suffer from that complication before treatment (blue) or no, they did not (red) are shown in the bar labelled by that complication (axis), for each complication in the list. **(B)** Participants were asked if they suffered from each of a list of known sickle cell disease complications after they began their hydroxyurea treatment. The proportion of participants out of the entire study group who answered yes, they did suffer from that complication during treatment (blue) or no, they did not (red) are shown in the bar labelled by that complication (axis), for each complication in the list.

We considered whether HU was able to reduce the number of severe complications to sickle cell disease the participant experienced and whether it had serious side effects. In the first case, each participant was asked whether they suffered from a range of known complications of sickle cell disease at two different time points; before they began HU treatment, and during their treatment. For each complication, and for each time point, the participants answered either yes, they did experience that complication at that time, or no, they did not. We then defined a score for each patient at each time point by counting the number of complications each patient claimed to experience. We ran a *t*-test for these scores and found the *p*-value of obtaining them given that there was no systematic difference in the population between the two-time points, before or during treatment, was less than 0.001. We found evidence to suggest that HU reduced complications of sickle cell disease in this population. Similarly, 347 (92.5%) participants had less frequency of vaso-occlusive crises per year, 173 (84.8%) had fewer transfusion needs, and 318 (84.6%) had fewer hospital admissions per year (Table 4). On the other hand, in the second case, 92 of 378 participants (24.3%) claimed to experience side effects of HU while 194 (51.3%) participants reported none to minimal side effects using HU. We also found that the packed cell volume (PCV) of patients greatly improved while using HU (Table 4; Figure 4). We found some evidence to support that exceptionally high HU doses were prescribed to patients with exceptionally high frequencies of painful episodes (Figure 5). Furthermore, patients with such high frequencies of painful episodes before the commencement of HU used doses as much as 35 mg/kg/d. They also reported a reduction in those painful crises since the commencement of HU (Table 4). Finally, we saw good visual evidence in this study that HU reduces the complications of sickle cell disease dramatically, except for bone pain (Figure 6A and Figure 6B).

**TABLE 4:** Experience with the use of hydroxyurea

Experience	Before using HU n (%)	Currently using HU n (%)
Number of VOC per year	—	—
0–1	47 (12.5)	240 (63.5)
2–3	140 (37)	92 (24.4)
4–5	106 (28.1)	29 (7.7)
6–7	29 (7.7)	4 (1.1)
>7	53 (14)	9 (2.5)
Number of hospital admission per year	—	—
0–1	161 (42.6)	308 (81.5)
2–3	129 (34.1)	50 (13.2)
4–5	57 (15.1)	15 (4.0)
6–7	13 (3.5)	1 (0.3)
>7	16 (4.4)	1 (0.3)
Number of transfusion	—	—
0	123 (32.5)	260 (68.8)
1–3	187 (49.5)	77 (20.4)
>3	45 (11.9)	15 (4.0)
PCV%	—	—
<20	36 (21.4)	8 (4.8)
20–25	106 (63.1)	64 (38.1)
>25	26 (15.5)	96 (57.1)
Minimum PCV (Min)	14%	18%
Maximum PCV (Max)	39%	39%

### Statistical Models of Patient Responses

The participants were asked whether they thought HU is affordable, easily available, has been prescribed by their doctor, and has no severe side effects. We performed a Pearson’s Chi-squared test with Yates’ continuity correction for the responses to each question, wherein each case the null hypothesis was members of the two groups (those currently using HU, and those not currently using HU) are equally likely to say “yes” to the question. For the question on affordability, we found no strong evidence to reject the null hypothesis that a patient who finds HU expensive is just as likely to use the drug as one who does not (chi-squared = 2.7991, df = 1, *p*-value = 0.09). For the question on availability, we found strong evidence to reject the null hypothesis that a patient who finds HU more easily available will be just as likely to use the drug (chi-squared = 15.415, df = 1, *p*-value = 9e-05). For the question on side effects, we found no evidence to reject the null hypothesis that a patient who has no severe side effects will be as likely to use the drug (chi-square = 0.41891, df = 1, *p*-value = 0.5). For the question on prescription, the proportion of participants who had not been prescribed the drug by their doctor (whether they currently use it or not) was too small for a chi-square test to be valid.

Also, we fit logistic regression models to the same data. In each model we constructed, the predictor was the response to one of the questions described above (affordable, available, prescribed by their doctor, and no severe side effects) and in each case, the outcome chosen was the same: is the patient currently using the drug (where “success” is defined as a “yes” response). We fit models for crude odds ratios (without covariates) and separately for adjusted odds ratios where the covariates were gender, age, and hospital location. Below we quote the odds ratios as log odds for both the crude and adjusted models.

For the question on affordability, we found no significant association in the crude model or the model where we adjusted for our chosen covariates (age, gender, and hospital location). For the question on availability, we found the crude log odds of a patient currently using HU, given that they find HU easily available, was 1.4 ± 0.3 (*p*-value: 9.43e-05) over a patient who does not find it easily available. After adjusting for gender, age, and hospital location, this became a log-odds of 0.5 ± 0.4 (*p*-value: 0.001). For the question on prescription, we found the crude log odds of a patient currently using HU, given that it has been prescribed by their doctor, was 1.7 ± 0.5 (*p*-value: 0.0005) over a patient who has not had the drug prescribed. After adjusting for the selected covariates, the log odds became 2.9 ± 0.8 (*p*-value. For the question on side effects, we found no association, for the crude model, between patients currently using HU, and patients who experience HU side effects. However, after adjusting for the covariates, the log odds of a patient currently using HU, given that they do not experience side effects of the drug, became –1.9 ± 0.5 (*p*-value: 7e-6) over a patient who does experience side effects.

## Discussion

Studies have shown the efficacy of HU in the management of sickle cell disease. HU improves numerous adverse events resulting from the complications of SCD including severe painful episodes, anaemia, hospitalization and transfusion frequencies (Charache et al., 1996; Ware, 2010; Thornburg et al., 2010; Cunningham-Myrie et al., 2015). The findings of this study equally showed these benefits as participants reported a reduced need for blood transfusion, hospital admissions and annual painful episodes. We found that the mean start dose of HU for our participants was 18 mg/kg/d which complies with earlier studies that recommend initiating HU at doses not more than 20 mg/kg/d. Similarly, our study found that high doses of HU were prescribed to patients with exceptionally high frequencies of painful crisis. These patients with high frequencies of painful episodes used HU up to 35 mg/kg/d since the commenced treatment of SCD using HU therapy. The PCV count of most patients increased after the commencement of HU compared with the values before the patient started using the drug. This finding agrees with earlier studies that support an increase in PCV count as a result of HU therapy (McGann and Ware, 2011; Smith et al., 2011; Heeney and Ware, 2010; Charache et al., 1995). A medication that significantly reduces the severity and frequency of painful crises, hospital admissions and need for blood transfusion among SCD patients is worth recommending.

This study has also demonstrated that most participants, whether they used the drug or had discontinued, felt the drug was expensive. Other than HU being expensive for most patients in this study, many of those who discontinued HU reported inaccessibility as a factor. These factors have been reported by Adeyemo et al., as a major barrier to the use of HU in Nigeria. The study went ahead to show that nearly all participants had been recommended on HU therapy by their doctors. The majority of participants represented also claimed that they do not suffer from severe side effects using HU therapy. However, HU availability potentially explained at least some of the variation in the cohort concerning HU use. Those who used HU in this study could access it easily, while those that had stopped did not have easy access. We also found that 306 (90.3%) patients who were using HU and 16 (41.0%) patients who had discontinued the use of the drug would recommend it to other patients.

There are several unique aspects of this study that are worth noting. Firstly, the diversity of the patient population, which would be crucial for generalizing the perception and experience with the use of HU treatment in different Nigerian settings. Also, the location of the five tertiary institutions across three geopolitical zones in Nigeria. These sites reported the highest number of patients using HU among the 20 participating hospitals in the SPARCO registry. Lastly, the patients’ responses in the study were corroborated with their medical reports as recorded in the registry.

## Conclusion

In this study, we were able to demonstrate that patients significantly had a good experience with the use of HU, minimal side effects while using HU, fewer pain crises per year as a result of the therapy, less need for blood transfusion, improved PCV and fewer annual hospital admissions. We were able to also deduce that a participant who finds HU easily accessible is more likely to use HU treatment than a participant who does not. We found that majority of participants felt that HU was expensive whether they used it or not, and that doctors routinely prescribe HU to their patients, whether it is easily available or not. Most patients make out-of-pocket payments for medications, while a few of them are covered by the national insurance scheme. This can be financially burdensome considering that this is a medication expected to be taken every day for the rest of their lives. Government intervention is needed to subsidize the drug or provide them free of charge as can be obtained in some climes. This can be achieved if resources are made available through a collaboration of the government and the indigenous companies manufacturing HU. We can conclude that our study has shown that patients had a positive outcome using HU by demonstrating that HU is effective in reducing the frequency of vaso-occlusive crises, need for blood transfusions, and hospitalizations among SCD patients who have reported the use of HU in the SPARCO Nigerian registry. It has also been shown that patients themselves would recommend the drug whether they were using it or not. We can therefore say that HU is safe among the study participants. The findings of this study may increase the use of hydroxyurea in Nigeria if the major barriers of cost and access to hydroxyurea are addressed on a national level.

## Limitation

This study was designed to take responses from patients via phone as such, information such as patients current weight and Packed Cell Volume (PCV) count was available for only patients who had visited the clinic recently and/or had checked the value a day or two before the interview. Similarly, MCV and HbF values were not collected since it goes beyond the scope and design of this study.

## Data Availability

The raw data supporting the conclusion of this article will be made available by the authors, following the approval of the funders.
